# Protective effect of chronic administration of pelargonidin on neuronal apoptosis and memory process in amyloid-beta-treated rats

**DOI:** 10.22038/AJP.2021.17680

**Published:** 2021

**Authors:** Nazita Alisavari, Sara Soleimani-Asl, Mohammad Zarei, Nasrin Hashemi-Firouzi, Siamak Shahidi

**Affiliations:** 1 *Department of Physiology, School of Medicine, Hamadan University of Medical Sciences, Hamadan, Iran*; 2 *Department of Anatomy, School of Medicine, Hamadan University of Medical Sciences, Hamadan, Iran*

**Keywords:** Alzheimer’s disease, Hippocampus, Learning and memory, Pelargonidin, Apoptosis

## Abstract

**Objective:**

Alzheimer's disease (AD) is a progressive neurodegenerative disorder associated with impaired cognitive skills and learning and memory dysfunctions.  It has been suggested that pelargonidin (PG), as an antioxidant agent, has a neuroprotective effect. PG could prevent damaging effects of amyloid-beta (Aβ) deposition. The aim of this study was to determine the chronic effect of PG on hippocampal neurons and memory processes in a rat model of AD.

**Materials and Methods:**

Twenty-eight male adult rats were divided into sham, AD, AD+PG (5 μg, intracerebroventricular), and PG (5 μg, intracerebroventricular) groups. Intracerebroventricular (ICV) injection of Aβ peptides (6 μg) was done using stereotaxic surgery. ICV administration of PG or saline was performed daily for 28 consecutive days. Behavioral analysis was performed using the novel object recognition (NOR) and passive avoidance tests. Neuronal apoptosis was detected using TUNEL assay in the hippocampus.

**Results:**

The ICV injection of Aβ reduced step-through latency and discrimination index in behavioral tests (p<0.001). Aβ increased the number of apoptotic neurons (p<0.001). PG treatment decreased the time spent in the dark compartment and neuronal apoptosis in the AD+PG rats (p<0.001). PG increased the discrimination index in the NOR test (p<0.001). Although PG did not change behavioral variables, it decreased cell death in the PG group.

**Conclusion:**

PG attenuated neuronal apoptosis and improved cognition and memory deficiency in AD rats. The protective effect of PG against Aβ may be due to its anti-apoptotic property. It is suggested that PG can be useful to treat AD.

## Introduction

Alzheimer's disease (AD) is the most common type of dementia and is characterized by the accumulation of amyloid-beta peptides (Aβ) (Berrios, 1990 ▶; Jack et al., 2011 ▶). AD is a progressive neurodegenerative disorder associated with impaired cognitive skills and learning and memory dysfunctions (Dringenberg, 2000 ▶). Abnormal amyloid precursor protein (APP) processing, oxidative stress, inflammation, and factors can induce a decline in mental ability and changes in behavior (Fares and Borrmann, 2018 ▶; Shen et al., 2006 ▶; Sultana et al., 2006 ▶).

Aβ peptides promote oxidative stress (Butterfield et al., 2013 ▶) by enhancing the production of free radicals in AD (Butterfield et al., 2013 ▶; Pohanka, 2014 ▶; Swomley et al., 2014 ▶).  The antioxidant system possibly prevents or neutralizes the damaging effects of free radicals (Pham-Huy, 2008 ▶). The efficiency of the antioxidant defense systems is decreased during AD leading to free radical generation in the brain (Chen and Zhong, 2014 ▶). High levels of antioxidants have been suggested useful in the treatment of AD (Chen and Zhong, 2014 ▶). 

Despite developments in the knowledge on the pathophysiology of AD, there is no effective cure for it. Aβ proteins may be a protective response to oxidative stress (Chen and Zhong, 2014 ▶; Pohanka, 2014 ▶; Swomley et al., 2014 ▶), Furthermore, the effectiveness of antioxidants against oxidative stress has been widely considered. Anthocyanins are water-soluble pigments found in plant tissues (Wrolstad, 2004 ▶). Pelargonidin (PG), a member of the anthocyanin group, is a natural bioactive agent that is responsible for the red color in plants (Fang, 2015 ▶). 

PG possesses a potent antioxidant capacity, prevents cellular oxidative stress (Karthi et al., 2017 ▶; Xu et al., 2018 ▶), and has a beneficial effect on inflammation, hyperglycemia, and memory (Duarte et al., 2018 ▶; Mirshekar et al., 2010 ▶; Mirshekar et al., 2011 ▶; Xu et al., 2018 ▶). It was suggested that PG could delay the onset or progression of AD (Sohanaki et al., 2016 ▶). PG crosses the blood-brain barrier (Youdim et al., 2003 ▶), exerted a neuroprotective effect in 6-hydroxydopamine rat model of hemi-parkinsonism (Roghani et al., 2010 ▶), and improved memory impairment (Mirshekar et al., 2011 ▶; Roghani et al., 2010 ▶; Sohanaki et al., 2016 ▶). 

Besides, it was suggested that anthocyanins could reduce cell death during oxidative stress conditions in neurodegenerative diseases (Ereminas et al., 2017 ▶). It has a protective effect against DNA damage induced by acetaminophen (Seo et al., 2020 ▶) and chemotherapeutic drugs in mice (Khandelwal and Abraham, 2014 ▶). 

No evidence is available about the chronic therapeutic effects of PG against neuronal apoptosis in AD. This study investigates the preventive effect of PG on Aβ1-42-induced neuronal death and learning and memory impairment in a rat model of AD. 

## Materials and Methods

**Animals**

In this study, 28 adult male Wistar rats weighing 250-300 g (prepared from the animal house of Hamadan University of Medical Sciences) were used. The animals were maintained in a room with temperatures ranging from 20 to 24°C under a 12 hr light/dark cycle. They had free access to the standard food pellets and water. All experimental procedures were approved by the Ethics Committee of the Hamadan University of Medical Sciences (1393.6381) and performed according to the Guide for Care and Use of Laboratory Animals published by the United States National Institutes of Health (NIH Publication No. 85-23, revised 1985).

**Cannula implementation and Intracerebroventricular (ICV) injection of Aβ and PG**

Rats were anesthetized by a mixture of ketamine and xylazine (100/10 mg/kg) and placed in a stereotaxic apparatus (Stoelting, USA). A guide cannula was lowered into the right lateral ventricle using the following coordinates: 0.9 mm posterior to the bregma, 1.5 mm lateral to the sagittal suture, and 3.2 mm ventral to the skull surface (Paxinos). The guide cannula was secured using two stainless steel screws anchored to the skull with dental acrylic cement. At the end of the surgery, the skin was sutured and the animals were individually housed and allowed to recover.

Aβ1-42 (Tocris, UK) peptide was solubilized in dimethyl sulfoxide and normal saline (0.9%). Then, the aliquots of Aβ1-42 were prepared at a concentration of 6 μg/μl and preserved at -70°C until its use. Intracerebroventricular (ICV) injection was performed via a 30-gauge injector cannula (1 mm below the tip of the guide cannula) with a Hamilton syringe (Hamilton Laboratory Products, Switzerland) attached to the injector cannula by polyethylene micro-tubing (PE-20). The rats were subjected to an ICV microinjection of Aβ or its vehicle. ICV injection of Aβ can induce AD in rodents (Shahidi et al., 2018).

One day following the injection of Aβ, rats were treated with daily ICV injections of either PG (5 μg/μl, Tocris, UK) or its vehicle for 28 consecutive days until the brain was removed for histological assessment. A single dose was estimated and determined according to the effective single dose of PG in the previous reports (Mirshekar et al., 2011; Soleimani Asl et al., 2019b). PG was dissolved in ethanol (60%) and further diluted in normal saline. It was then stored at -20^°^C before administration.

ICV injection is used for direct administration of a smaller amount of substances into the cerebrospinal fluid in cerebral ventricles; via this method, drugs can cross the blood-brain barrier with no barriers to limit drug delivery into the brain. Therefore, drugs can enter several parts of the brain, such as the hippocampus and cortex. ICV administration of drugs only affects brain structures with no peripheral effects.

**Experimental groups**

 The rats were randomly divided into four groups: (1) Sham group, which received saline (6 μl) under surgery, followed by receiving saline (2 μl); (2) AD group, which received Aβ (6 μg) and it was subjected to surgery and treated with saline (2 μl); (3) AD+PG group, which received Aβ and it was subjected to surgery, followed by PG (5 μg); and (4) PG group, which received 6 μl saline via ICV injection under surgery, followed by PG (5 μg).

**Figure 1:**
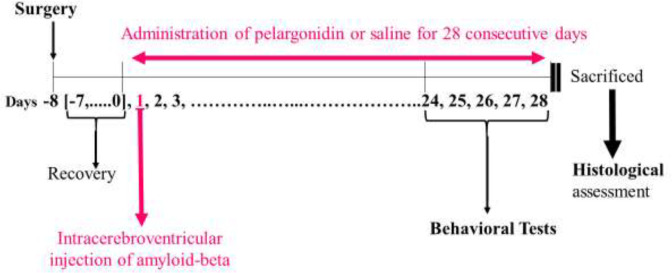
Experimental design

The Aβ, saline, and PG ICV microinjections were done via a 30-gauge injector cannula with a Hamilton syringe (Hamilton Laboratory Products, Switzerland). Daily treatment of saline or PG was performed for 28 consecutive days. During behavioral tests, treatments were continued (Figure 1).

**Novel Object Recognition (NOR) test**

The NOR test was performed to evaluate the cognition behavior in animals. This cognition behavior arises from cortical functioning (Shahidi et al., 2018). The apparatus consists of a brown wooden open box (48 ×42 ×36 cm). On the first day, the rats were given one habituation session in the arena without any object (5 min). On the second day, the training phase was performed, in which, two similar objects were placed close to (10 cm) two adjacent corners of the arena. Then, the rats were placed in the middle of the box and allowed to explore the two objects. The rat behavior was recorded by a video camera system for 10 min. 

The exploration process of an object was defined as smelling the object. On the third day, in the retention test, one of the objects was replaced with a novel object and the rats were placed back in the open field for 10 min. The rat response to the novel object, the discrimination index, was assessed by subtracting the mean exploration time of the familiar object from the mean exploration time of the novel object.

**Passive avoidance learning (PAL) apparatus**

The apparatus and procedure were the same as described in our previous studies (Shahidi et al., 2018). Briefly, first, the rats were subjected to two trials (with an interval of 30 min) to habituate to the apparatus. Then, they were trained for passive avoidance learning by placing them in the light compartment.

At first, the rats entered into a light compartment of the apparatus and 5 sec later, the guillotine door was opened. Because of the natural tendency of the rats to the dark environment, they tend to enter the dark compartment. The door was closed after the entrance of the rats into the dark compartment and after 30 sec, they were taken from the dark compartment and placed in their cage. After 30 min, this trial was repeated. The measurement of the entrance latency to the dark compartment (step-through latency in the acquisition trial, STLa) was carried out after the rats completely entered the dark compartment.

 Then, the door was closed, an electrical shock was delivered (0.5 mA) for 2 sec, and the rat was returned to its cage after 30 sec. This procedure was repeated again after 2 min. For the next stages, the foot shock was delivered after the rat reentered the dark and had placed all four paws in the dark compartment. Finally, the training trial was terminated when the animals remained continuously in the bright compartment for 120 sec and the number of entries into the dark chamber to acquisition was recorded.

**Retention test**

Exactly 24 hr after performing the PAL acquisition trial, the retention test was performed. In this step, like the PAL training trial, the rat was placed in the light chamber and after 5 sec, the guillotine door was opened in order to start the recording process of the step-through latency during reacquisition (STLr) and the time spent in the dark compartment (TDC) for up to 300 sec was recorded. The retention test was terminated when the rat did not enter the dark chamber within 300 sec, and a ceiling score of 300 sec was recorded.

**Tissue preparation and TUNEL staining**

The day after the behavioral assessments, the rats were deeply anesthetized by ketamine (100 mg/kg) and xylazine (10 mg/kg) and transcardially perfused with paraformaldehyde. The brains were removed from the skulls and fixed in the fixation solution. Then, coronal sections (5 μm) of the hippocampus were serially prepared by a microtome (Leica, IL, USA). After deparaffinization and rehydration, the alternate sections were stained using TdT-mediated dUTP nick-end labeling (TUNEL) kit. TUNEL staining was performed to detect apoptotic cells. 

TUNEL labels cells have fragmented DNA and sustained programmed cell death (Anderson et al., 2000). The TUNEL staining was performed using a TUNEL kit (Roche) according to the manufacturer’s instructions as previously described (Hashemi-Firouzi et al., 2017; Pourheydar et al., 2016). The apoptotic neurons in the hippocampal cornu ammonis 1 (CA1) layer were analyzed under a light microscope (BX40, Olympus). The cells that clearly displayed dark brown colored particles in the nucleus were defined as apoptotic neurons.

**Statistical analysis**

The Kolmogorov–Smirnov test was used to analyze the normality of the data. The one-way analysis of variance (ANOVA) followed by the Tukey *post-hoc* test were applied to determine the statistical significance of differences among experimental groups. The non-parametric Kruskal-Wallis test was used to assess the significance among variables for non-normality of data. A p<0.05 was considered significant. Data are presented as mean±S.E.M.

## Results

**Effect of PG on NOR test**

**Figure 2:**
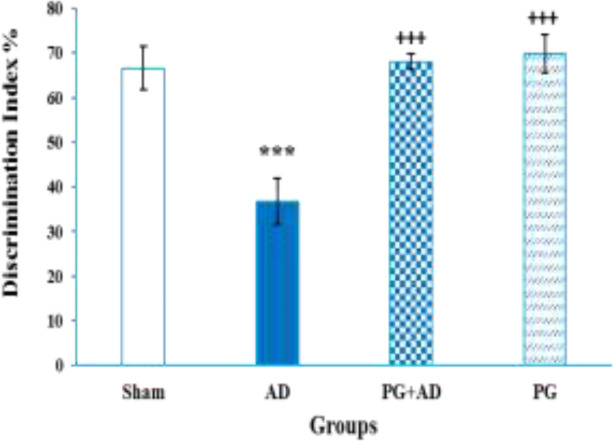
Effect of chronic treatment with pelargonidin on discrimination index in object recognition test. ***p<0.001 compared with the control group and +++p<0.001, PG+AD group and PG+AD groups compared with AD group. (n=7 per group). Each column represents mean±SEM

The Kolmogorov–Smirnov test demonstrated the normality of data of the NOR test (p>0.05). The one-way ANOVA showed a significant difference in the discrimination index between the groups (F (3, 27) =14.148, p<0.001; Figure 2). The Tukey *post-hoc* test showed that the discrimination index of AD rats was significantly lower than that of the sham, AD+PG, and PG rats (p<0.001). There were no significant differences among the control, sham, AD+PG, and PG groups. 

**Effect of PG on PAL test**

The Kolmogorov–Smirnov test showed the non-normality of data for PAL test in some groups; therefore, the nonparametric Kruskal–Wallis test was used to assess the significant difference among groups regarding the STLa (p<0.05), number of trials (p<0.001), STLr (p<0.001), and TDC (p<0.001). 

**Figure 3:**
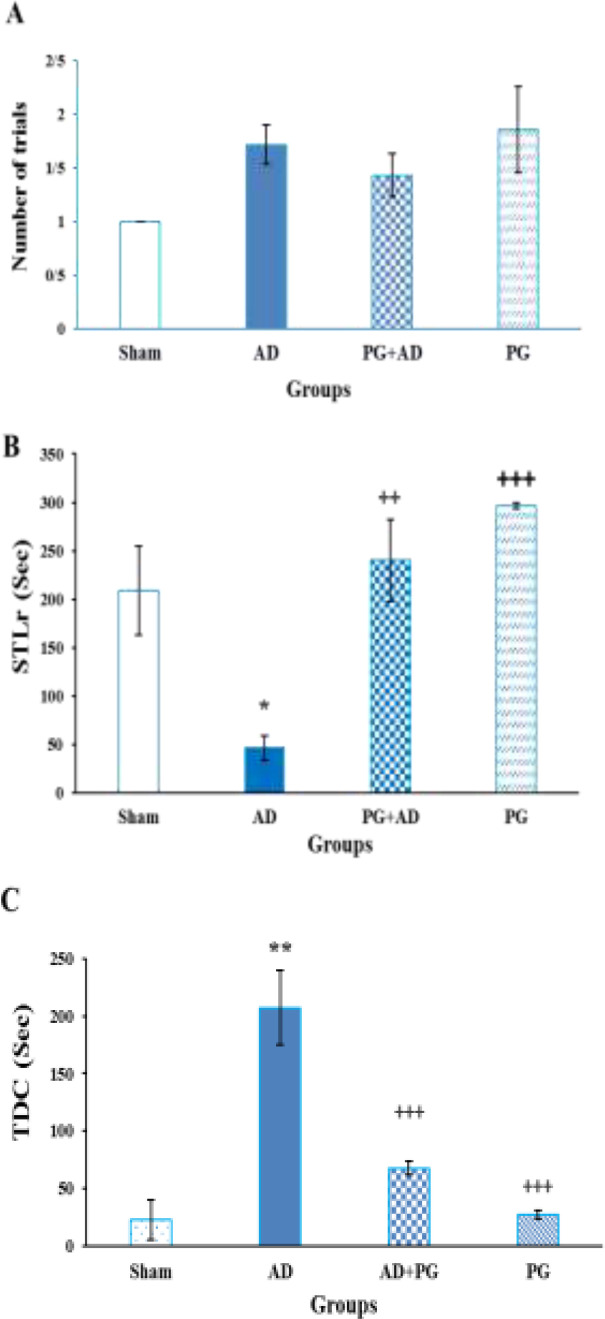
Effect of chronic treatment with pelargonidin on the number of trials to acquisition (A), step-through latency in the retention test (B), and time spent in the dark compartment (C) in the passive avoidance learning task. **p<0.01 and *p<0.05 compared with the sham group and +++p<0.001 and ++p<0.01, PG+AD group and PG+AD groups compared with AD group. (n=7 per group). Each column represents mean±SEM

The Kruskal-Wallis test revealed no significant differences among groups in the STLa (F (3, 28=7.532.148, p=0.057). This result indicated that there was no significant difference in the exploratory behavior of the animals to enter the dark compartment. In addition, The Kruskal–Wallis test showed that there was no significant difference in the number of trials among groups [F (3, 28) =7.676, p=7.676, Figure 3a]. 

The Kruskal–Wallis test indicated a significant difference in the STLr among the groups [F (3, 28) =13.074, p<0.01, Figure 3b]. Pairwise comparisons showed that the STLr in the AD group was significantly lower than the sham, AD+PG, and PG groups (p<0.05, p<0.01, and p<0.001, respectively). 

The statistical analysis by Kruskal–Wallis test indicated a significant difference in TDC among the animals (F (3, 28) =17.144, p<0.001; Figure 3c). Pairwise comparisons of the groups showed that the TDC of the AD group was significantly more than the sham, AD+PG, and PG groups (p<0.01, p<0.001, and p<0.001, respectively). However, there was no significant difference in the TDC among the sham, AD+PG, and PG groups (p>0.05).

**Effects of PG on hippocampal apoptotic cells**

**Figure 4:**
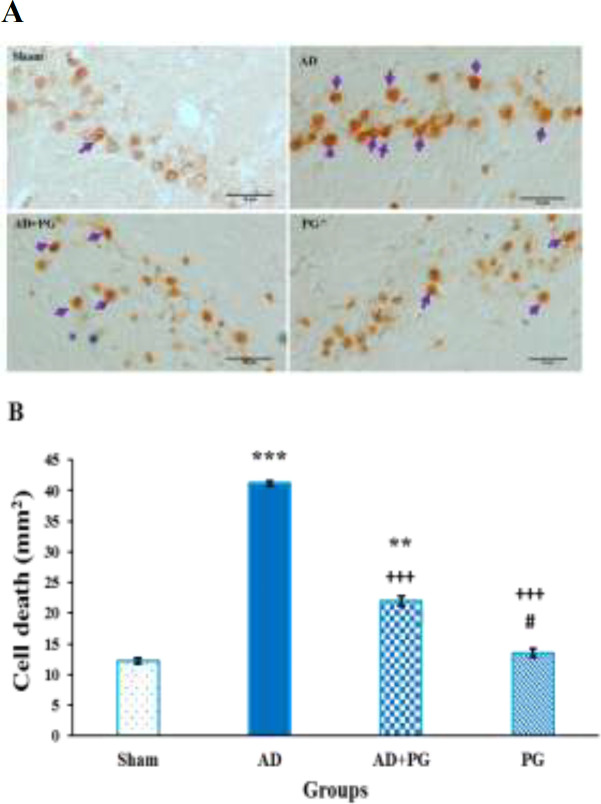
Light micrographs of neuronal apoptosis in the hippocampal CA1 area; (A) Sections derived from the groups stained by TUNEL. Blue arrows show apoptotic neurons. Scale bar=100 μm, magnification: ×400. (B) The number of apoptotic neurons (cells clearly displaying dark brown colored particles in the nucleus) was calculated. ***p<0.001 and **p<0.01 compared to the sham group; +++p<0.001 compared with the AD group; and #p<0.05 compared with the AD+PG group. (n=7 per group). Each column represents mean±SEM

Figure 4 shows the hippocampal neuronal sections stained using the TUNEL kit. The Kolmogorov–Smirnov test showed the non-normality of data for apoptotic cells (p<0.05). Statistical analysis by Kruskal–Wallis test showed that there was a significant difference between groups (F (3, 28) =21.421, p<0.001, Figure 4b). Pairwise comparisons of the groups showed that the AD group had significantly more apoptotic neurons than the sham, AD+PG, and PG groups (p<0.001). The AD+PG rats had more apoptotic neurons compared with the sham (p<0.01) and PG (p<0.05) groups. 

## Discussion

The results of our study showed that PG, a natural flavonoid, attenuated Aβ-induced neuronal apoptosis, and memory deficits in AD rats. The ICV injection of Aβ induced neuronal apoptosis in the hippocampus that is linked to deficits in memory.

In the present study, the TUNEL assay results revealed that Aβ injections induced neuronal death via apoptosis in the hippocampus. It has been found that Aβ can induce neurotoxicity, which leads to cell death in the hippocampus (Chen and Zhong, 2014). In addition, Aβ induced learning and memory deficiency after the intrahippocampal or ICV injection of Aβ peptide in animals (Facchinetti et al., 2018; Karthick et al., 2019; Kim et al., 2016). In addition, Aβ toxicity leads to neuronal cell death in the hippocampus (Chen and Zhong, 2014). Aβ can reduce antioxidant defense response and induce overproduction of reactive oxygen species (ROS) (Butterfield et al., 2013) leading to neuron loss in the brain (Shen et al., 2006) and cognitive deficits in AD (Lovell and Markesbery, 2007). 

Using antioxidants is one of the most common therapeutic approaches for the treatment of memory impairment (Lee et al., 2010). The red pigment PG belongs to the anthocyanins and is found in plants (Fang, 2015). It can cross the brain-blood barrier (Youdim et al., 2003). The results of the present study demonstrated that the chronic ICV microinjection of PG exerts protective effects against Aβ-induced memory impairment in rats. It has been reported that PG has a neuroprotective effect against 6-hydroxydopamine toxicity in animal models (Roghani et al., 2010). In addition, acute treatment with PG could improve memory deficit caused by Aβ (Sohanaki et al., 2016; Soleimani Asl et al., 2019a) and chronic treatment with PG restored the activity of some antioxidant enzymes and recall capability in diabetic rats (Mirshekar et al., 2011).

Anthocyanin, as a group of flavonoids, prevented the generation of free oxygen radicals (Corona and Vauzour, 2017; Ereminas et al., 2017; Fang, 2015) and inhibited the induction of cell apoptosis (Khandelwal and Abraham, 2014; Shih et al., 2005). Flavonoids, such as PG inhibited oxidative stress, inflammation response, and neuronal apoptosis (Cai et al., 2020) through the enhancement of an antioxidant defense system (Ereminas et al., 2017). Aβ administration induced a reduction in antioxidant capacity (Durán-González et al., 2013). PG treatment increased the antioxidant level of the hippocampal neurons (Mirshekar et al., 2010), inhibited the ROS-induced inflammatory apoptotic response (Seo et al., 2020) and controlled the signals responsible for DNA methylation (Karthi et al., 2017). The protective effect of PG may be due to its anti-apoptotic property. However, in this study, we did not investigate the expression of protein markers of neuronal apoptosis. 

Another finding of this study was that PG increased reacquisition of the passive avoidance memory in healthy rats. It has been reported that PG can enhance learning and memory (Soleimani Asl et al., 2019a). There is a steady-state balance between the production and removal of oxidant agents in normal conditions (Calabrese et al., 2000). PG enhanced memory by potentiating the antiapoptotic mechanism in the hippocampus. It enhanced the antioxidant defense system (Ereminas et al., 2017), restored the hippocampal antioxidant capacity (Soleimani Asl et al., 2019a) and potentiated the antiapoptotic mechanism (Seo et al., 2020). Accordingly, through these mechanisms, it can enhance the memory reacquisition in normal conditions. However, we did not assess the levels of antioxidant or oxidant biomarkers in the hippocampus.

In conclusion, PG attenuated neuronal apoptosis and improved cognition and memory deficiency in AD rats. The ICV injections of Aβ impaired cognition, learning, and memory through enhancing the apoptosis in hippocampal neurons. The protective effect of PG against Aβ may be due to its anti-apoptotic property. Therefore, PG may be useful to treat AD through antioxidant activity and inducing a decrease in neuronal apoptosis against Aβ in the brain. Feature investigations are necessary to have a better understanding of these effects. 

## References

[B1] Anderson AJ, Stoltzner S, Lai F, Su J, Nixon RA (2000). Morphological and biochemical assessment of DNA damage and apoptosis in Down syndrome and Alzheimer disease, and effect of postmortem tissue archival on TUNEL. Neurobiol Aging.

[B2] Berrios GE (1990). Alzheimer's disease: a conceptual history. Int J Geriatr Psychiatry.

[B3] Butterfield DA, Swomley AM, Sultana R (2013). Amyloid beta-peptide (1-42)-induced oxidative stress in Alzheimer disease: importance in disease pathogenesis and progression. Antioxid Redox Signal.

[B4] Cai Y, Li X, Pan Z, Zhu Y, Tuo J, Meng Q, Dai G, Yang G, Pan Y (2020). Anthocyanin ameliorates hypoxia and ischemia induced inflammation and apoptosis by increasing autophagic flux in SH-SY5Y cells. Eur J Pharmacol.

[B5] Calabrese V, Bates TE, Stella AM (2000). NO synthase and NO-dependent signal pathways in brain aging and neurodegenerative disorders: the role of oxidant/antioxidant balance. Neurochem Res.

[B6] Chen Z, Zhong C (2014). Oxidative stress in Alzheimer's disease. Neurosci Bull.

[B7] Corona G, Vauzour D (2017). Neuroprotective effects of phytochemicals in neurological disorders.

[B8] Dringenberg HC (2000). Alzheimer's disease: more than a 'cholinergic disorder' - evidence that cholinergic-monoaminergic interactions contribute to EEG slowing and dementia. Behav Brain Res.

[B9] Duarte LJ, Chaves VC, Nascimento M, Calvete E, Li M, Ciraolo E, Ghigo A, Hirsch E, Simoes CMO, Reginatto FH, Dalmarco EM (2018). Molecular mechanism of action of Pelargonidin-3-O-glucoside, the main anthocyanin responsible for the anti-inflammatory effect of strawberry fruits. Food Chem.

[B10] Durán-González J, Michi ED, Elorza B, Perez-Córdova MG, Pacheco-Otalora LF, Touhami A, Paulson P, Perry G, Murray IV, Colom LV (2013). Amyloid β peptides modify the expression of antioxidant repair enzymes and a potassium channel in the septohippocampal system. Neurobiol Aging.

[B11] Ereminas G, Majiene D, Sidlauskas K, Jakstas V, Ivanauskas L, Vaitiekaitis G, Liobikas J (2017). Neuroprotective properties of anthocyanidin glycosides against H2O2-induced glial cell death are modulated by their different stability and antioxidant activity in vitro. Biomed Pharmacother.

[B12] Facchinetti R, Bronzuoli MR, Scuderi C (2018). An animal model of Alzheimer disease based on the intrahippocampal injection of amyloid beta-peptide (1-42). Methods Mol Biol.

[B13] Fang J (2015). Classification of fruits based on anthocyanin types and relevance to their health effects. Nutrition.

[B14] Fares A, Borrmann D (2018). Neurochemical aspects of Alzheimer's disease and movement disturbances: A theory of beta-amyloid and tau-protein. Am J Alzheimers Dis Other Demen.

[B15] Hashemi-Firouzi N, Komaki A, Soleimani Asl S, Shahidi S (2017). The effects of the 5-HT7 receptor on hippocampal long-term potentiation and apoptosis in a rat model of Alzheimer's disease. Brain Res Bull.

[B16] Jack Jr CR, Albert MS, Knopman DS, McKhann GM, Sperling RA, Carrillo MC, Thies B, Phelps CH (2011). Introduction to the recommendations from the National Institute on Aging-Alzheimer's Association workgroups on diagnostic guidelines for Alzheimer's disease. Alzheimers Dement.

[B17] Karthi N, Karthiga A, Kalaiyarasu T, Stalin A, Manju V, Singh SK, Cyril R, Lee SM (2017). Exploration of cell cycle regulation and modulation of the DNA methylation mechanism of pelargonidin: Insights from the molecular modeling approach. Comput Biol Chem.

[B18] Karthick C, Nithiyanandan S, Essa MM, Guillemin GJ, Jayachandran SK, Anusuyadevi M (2019). Time-dependent effect of oligomeric amyloid-beta (1-42)-induced hippocampal neurodegeneration in rat model of Alzheimer's disease. Neurol Res.

[B19] Khandelwal N, Abraham SK (2014). Protective effects of common anthocyanidins against genotoxic damage induced by chemotherapeutic drugs in mice. Planta Med.

[B20] Kim HY, Lee DK, Chung BR, Kim HV, Kim Y (2016). Intracerebroventricular injection of amyloid-beta peptides in normal mice to acutely induce Alzheimer-like cognitive deficits. J Vis Exp.

[B21] Lee HP, Zhu X, Casadesus G, Castellani RJ, Nunomura A, Smith MA, Lee HG, Perry G (2010). Antioxidant approaches for the treatment of Alzheimer's disease. Expert Rev Neurother.

[B22] Lovell MA, Markesbery WR (2007). Oxidative DNA damage in mild cognitive impairment and late-stage Alzheimer's disease. Nucleic Acids Res.

[B23] Mirshekar M, Roghani M, Khalili M, Baluchnejadmojarad T, Moazzen SA (2010). Chronic oral pelargonidin alleviates streptozotocin-induced diabetic neuropathic hyperalgesia in rat: involvement of oxidative stress. Iran Biomed J.

[B24] Mirshekar M, Roghani M, Khalili M, Baluchnejadmojarad T (2011). Chronic oral pelargonidin alleviates learning and memory disturbances in streptozotocin diabetic rats. Iran J Pharm Res.

[B25] Paxinos G, Watson C (1998). The rat brain in stereotaxic coordinates.

[B26] Pohanka M (2014). Alzheimer s disease and oxidative stress: a review. Curr Med Chem.

[B27] Pourheydar B, Soleimani Asl S, Azimzadeh M, Rezaei Moghadam A, Marzban A, Mehdizadeh M (2016). Neuroprotective effects of bone marrow mesenchymal stem cells on bilateral common carotid arteries occlusion model of cerebral ischemia in rat. Behav Neurol.

[B28] Roghani M, Niknam A, Jalali-Nadoushan MR, Kiasalari Z, Khalili M, Baluchnejadmojarad T (2010). Oral pelargonidin exerts dose-dependent neuroprotection in 6-hydroxydopamine rat model of hemi-parkinsonism. Brain Res Bull.

[B29] Seo M, Kim H, Lee JH, Park JW (2020). Pelargonidin ameliorates acetaminophen-induced hepatotoxicity in mice by inhibiting the ROS-induced inflammatory apoptotic response. Biochimie.

[B30] Shahidi S, Asl SS, Komaki A, Hashemi-Firouzi N (2018). The effect of chronic stimulation of serotonin receptor type 7 on recognition, passive avoidance memory, hippocampal long-term potentiation, and neuronal apoptosis in the amyloid beta protein treated rat. Psychopharmacology (Berl).

[B31] Shen Y, He P, Zhong Z, McAllister C, Lindholm K (2006). Distinct destructive signal pathways of neuronal death in Alzheimer's disease. Trends Mol Med.

[B32] Shih PH, Yeh CT, Yen GC (2005). Effects of anthocyanidin on the inhibition of proliferation and induction of apoptosis in human gastric adenocarcinoma cells. Food Chem Toxicol.

[B33] Sohanak H, Baluchnejadmojarad T, Nikbakht F, Roghani M (2016). Pelargonidin improves memory deficit in amyloid beta 25-35 rat model of Alzheimer's disease by inhibition of glial activation, cholinesterase, and oxidative stress. Biomed Pharmacother.

[B34] Asl SS, Bergen H, Ashtari N, Amiri S, Łos MJ, Mehdizadeh M (2019a). Pelargonidin exhibits restoring effects against amyloid β-induced deficits in the hippocampus of male rats. Med J Islam Repub Iran.

[B35] Asl SS, Łos MJ, Mehdizadeh M (2019b). Pelargonidin improves amyloid β-induced deficits in the long-term potentiation in hippocampus of male rats. Physiol Pharmacol.

[B36] Sultana R, Perluigi M, Butterfield DA (2006). Protein oxidation and lipid peroxidation in brain of subjects with Alzheimer's disease: insights into mechanism of neurodegeneration from redox proteomics. Antioxid Redox Signal.

[B37] Swomley AM, Forster S, Keeney JT, Triplett J, Zhang Z, Sultana R, Butterfield DA (2014). Abeta, oxidative stress in Alzheimer disease: evidence based on proteomics studies. Biochim Biophys Acta.

[B38] Pham-Huy LA, He H, Pham-Huy C (2008). Free radicals, antioxidants in disease and health. Int J Biomed Sci.

[B39] Wrolstad RE (2004). Anthocyanin pigments—Bioactivity and coloring properties. J Food Sci.

[B40] Xu Y, Xie L, Xie J, Liu Y, Chen W (2018). Pelargonidin-3-O-rutinoside as a novel alpha-glucosidase inhibitor for improving postprandial hyperglycemia. Chem Commun (Camb).

[B41] Youdim KA, Dobbie MS, Kuhnle G, Proteggente AR, Abbott NJ, Rice‐Evans C (2003). Interaction between flavonoids and the blood-brain barrier: in vitro studies. J Neurochem.

